# Effect of brain radiotherapy strategies on prognosis of patients with *EGFR*-mutant lung adenocarcinoma with brain metastasis

**DOI:** 10.1186/s12967-021-03161-1

**Published:** 2021-11-30

**Authors:** Guangchuan Deng, Yingyun Zhang, Jiaojiao Ke, Qi Wang, Hongyue Qin, Jianbin Li, Zhenxiang Li

**Affiliations:** 1 Department of Radiation Oncology, Shandong Cancer Hospital and Institute, Shandong First Medical University and Shandong Academy of Medical Sciences, Jinan, 250117 China; 2 Shandong First Medical University and Shandong Academy of Medical Sciences, Jinan, People’s Republic of China; 3 Weihai Central Hospital, Weihai, People’s Republic of China

**Keywords:** EGFR-mutant, Lung adenocarcinoma, Brain metastasis, Lung-molGPA, Brain radiotherapy

## Abstract

**Purpose:**

Epidermal growth factor receptor (*EGFR*)-mutant lung cancers have a high risk of developing brain metastases (BM). Whole brain radiotherapy (WBRT), local radiotherapy, and WBRT + Boost are frequently used for treatment of BM. This retrospective study aimed to evaluate the difference in efficacy of these radiotherapy modes in patients with *EGFR*-mutant lung adenocarcinoma with BMs. Further, we determined the optimal radiotherapy regimen for patients based on Lung-molGPA.

**Methods and materials:**

We retrospectively enrolled 232 patients with *EGFR*-mutant lung adenocarcinoma with BMs. Patients were divided into three groups based on the different modes of brain radiotherapy: WBRT group, local radiotherapy group, and WBRT + Boost group. Graded prognostic assessment for lung cancer using molecular markers (Lung molGPA), overall survival (OS), and intracranial progression-free survival (iPFS) were calculated. Kaplan–Meier was used to compare iPFS and OS in different groups.

**Results:**

The median OS for the WBRT (n = 84), local radiotherapy (n = 65), and WBRT + Boost (n = 83) cohorts was 32.8, 59.1, and 41.7 months, respectively (*P* = 0.0002). After stratification according to the Lung-molGPA score, the median OS for the WBRT (n = 56), local radiotherapy (n = 19), and WBRT + Boost (n = 28) cohorts was 32.5, 30.9, and 30.8 months, respectively, in subgroup with score 1–2 (*P* = 0.5097). In subgroup with score 2.5–4, the median OS for the WBRT (n = 26), local radiotherapy (n = 45), and WBRT + Boost (n = 54) cohorts was 32, 68.4, and 51 months, respectively (*P* = 0.0041).

**Conclusion:**

The present study showed that in patients with *EGFR*-mutant lung adenocarcinoma with BM, local radiotherapy and WBRT + Boost perform similarly well both in the subgroups with low and high scores of Lung-molGPA. Considering the side effect caused by whole brain radiotherapy, we recommended local radiotherapy as optimal brain radiation mode for those subtype lung cancer patients.

## Introduction

Advanced lung adenocarcinoma is increasingly being treated with individualized molecular targeted therapy based on gene aberrations, and the mutant epidermal growth factor receptor (*EGFR*) is the most common therapeutic target [1–3]. For *EGFR*-mutant non-small cell lung cancers (NSCLC), brain metastasis (BM) is a severe complication in approximately 60% of patients during the course of the disease [4–6]. A previous study suggests that patients with BM have relatively low quality of life and shorter survival of only 3–6 months when untreated [7]. However, when treated with targeted therapy, patients with *EGFR*-mutant NSCLC with BM show median overall survival (OS) of 19 to 58 months [8, 9]. In patients with NSCLC harboring *EGFR* mutations, EGFR tyrosine kinase inhibitors (TKIs) exhibit some therapeutic efficacy against BM, however they show limited intracranial progression-free survival (iPFS) of 8 to 10 months [10, 11].

Brain radiotherapy, an effective therapeutic method, is critical in the treatment of brain metastases [12]. The most commonly administered modes of brain radiotherapy include whole brain radiotherapy (WBRT), local radiotherapy, and WBRT + Boost [13, 14]. Of these, WBRT is usually recommended for treatment of multiple BMs. However, WBRT can destroy the blood brain barrier (BBB) that greatly reduces the absorption of chemotherapy or targeted therapy and increases the concentration of EGFR TKIs in the cerebrospinal fluid, and also controls subclinical lesions. [12, 15–17]. Additionally, WBRT can worsen cognitive function and health-related quality of life [17, 18]. Radiotherapy targeting local metastases can reduce radiation damage to the surrounding normal brain tissue, and thus, reduces neurotoxicity. However, it can only target metastases in the radiation field, is limited to improve the control effect of multiple intracranial metastases, and therefore, is recommended only for a limited number of BMS (1 to 4) [19]. In contrast, WBRT + Boost offers advantages of both—it can control subclinical lesions with lower dose and increase the radiation dose to brain metastases as much as possible to destroy the local brain metastases [20, 21]. In recent years, some studies have suggested that WBRT + Boost is superior to WBRT in lung cancer patients with craniocerebral metastasis. However, those studies involved in mixed pathological type especially small cell lung cancer and did not take gene status into account to determine the therapy choice. [14, 20, 22].

The choice of brain radiotherapy mode should be based on the number of metastatic sites and overall condition of the patient. A recent study used graded prognostic assessment for lung cancer using molecular markers (Lung-molGPA) to evaluate patients according to age, Karnofsky Performance Status (KPS), extracranial metastasis, number of brain metastasis, and gene mutation status; a score of 0–4 indicated significant impact on OS in patients with NSCLCs with BMs, which may also influence the selection of radiotherapy mode [23, 24]. A study found that patients receiving local radiotherapy had an OS of 64 months (95% CI: 46 to not reach) with a better prognosis (DS-GPA, 2–4), while those receiving WBRT had OS of 52 months (95% CI 32 to 79) with the same prognosis (DS-GPA, 2–4) and those who received EGFR-TKI followed by RT at intracranial progression only had OS of 32 months (95% CI 26 to 39) [2].

Previous studies have showed that combining brain radiotherapy with EGFR TKIs can further increase local control of intracranial lesions. However, only few studies have explored the different outcomes in patients with *EGFR*-mutant lung cancer who received different radiotherapy modes, especially after considering the Lung-molGPA. Therefore, we performed this retrospective, real-world analysis for *EGFR*-mutant lung adenocarcinomas with BM to confirm the optimal brain radiotherapy regimen.

## Methods and materials

### Patient cohort

We screened > 800 patients diagnosed with NSCLC at our hospital between March 2008 and December 2019. The inclusion criteria were as follows: (1) pathologically diagnosed with primary lung adenocarcinoma; (2) mutation in *EGFR* exon 18, 19, or 21; (3) diagnosis of BM by enhanced computed tomography (CT) or magnetic resonance imaging (MRI); (4) detailed clinical information, including treatment options and clinicopathological features; (5) EGFR TKIs administered (e.g. gefitinib, erlotinib, or icotinib); (6) previously received brain radiotherapy, including WBRT, local radiotherapy, or WBRT + Boost; and (7) no other primary malignancies. Patients with incomplete medical records or those who failed to meet the above criteria were excluded from the study. The study was approved by the Ethics Committee of the of the Shandong Cancer Hospital, and was conducted in accordance with the Declaration of Helsinki.

The following characteristics were collected for analysis: age, sex, smoking history, *EGFR* mutation status, BM during initial diagnosis, and treatment. The start date of initial local therapies, start of EGFR-TKIs, intracranial progression, most recent follow-up, and death were recorded. Intracranial progression was defined as radiographic progression of preexisting BM, development of new BM, or both. OS was calculated from the date of pathological diagnosis of lung adenocarcinoma to date of death or reexamination at the last follow-up. Intracranial PFS was measured and reviewed at the last follow-up. The time to intracranial progression was calculated from the date of the start of brain radiotherapy to date of intracranial progression.

Lung-molGPA is a specific, graded prognostic assessment based on the patient's age, KPS, the number of extracranial and BM, and the status of gene mutations [25]. The scoring criteria are shown in Table 1.

**Table 1:** Lung-molGPA

Prognostic	0	0.5	1.0
Age (y)	≥ 70	< 70	NA
KPS	< 70	80	90–100
ECM	Present		Absent
Brain metastases, No. Gene status	> 4 EGFR neg/unk and ALK neg/unk	1–4 NA	NA EGFR pos or ALK pos

*GPA*: graded prognostic assessment, *ECM* extracranial metastases, *KPS* Karnofsky Performance Status, *NA* not applicable, *neg/unk* negative or unknown, *pos* positive

### EGFR genotyping

Genomic DNA was extracted from tissues obtained by fiberoptic bronchoscopy or puncture biopsy. In contrast, circulating tumor DNA (CtDNA) was isolated and purified from the blood. *EGFR* mutations were detected by next-generation sequencing (NGS), droplet digital polymerase chain reaction (ddPCR), or amplification refractory mutation system (ARMS)-PCR.

### Radiotherapy

In patients treated with whole brain radiotherapy, the median prescribed dose was 40 Gy (range, 30–50 Gy). The median prescribed dose for local radiotherapy was 50 Gy (range, 30–62.5 Gy). Additionally, in the WBRT + Boost group, the median prescription dose of whole brain was 40 Gy (range, 22–50 Gy). The median dose of the additional radiation boost for local metastases was 15 Gy (range, 6–50 Gy).

### Statistical methods

The characteristics of each group were descriptively compared, and the classified variables were tested by chi-square test. Kaplan–Meier method was used for survival analysis, and log rank test was used to test the influence of individual variables on survival. A *p* value < 0.05 (two-sided) was considered to be statistically significant. Analyses were performed using GraphPad Prism version 8.0.1.

## Results

### Patients characteristics

A total of 232 patients who met the inclusion criteria were enrolled in the study. According to the brain radiotherapy scheme, the patients were divided into three groups: WBRT, local radiotherapy, and WBRT + Boost. 84, 65, and 83 patients received WBRT, local radiotherapy, and WBRT + Boost, respectively. The characteristics of the patients are detailed in Table 2. The median age at diagnosis was 54 (range, 28–81) years. A majority of the patients were women (149, 64.2%) and non-smokers (182, 78.4%). As a first-line treatment, 46.6% of the patients (108/232) received EGFR TKIs, while 53.4% (124/232) received platinum-based treatment. More than 50% of the patients (62.1%) had craniocerebral metastasis at the time of initial diagnosis. Moreover, mutations in *EGFR* exons 19 and 21 were detected in 41.4% (96/232) and 49.6% (115/232) of the patients, respectively.

**Table 2:** Characteristics of 232 NSCLC patients and with chi-square test for categorical variables

Characteristics	WBRT (n = 84)	Local Radiotherapy (n = 65)	WBRT + Boost (n = 83)	*P* value
Age, years	53(28–77)	56(38–81)	53(33–78)	
> 60	21(25.0)	24(36.9)	23(27.7)	0.263
≤ 60	63(75.0)	41(63.1)	60(72.3)	
Sex				
Female	51(60.7)	43(66.2)	55(66.3)	0.703
Male	33(39.3)	22(33.8)	28(33.7)	
Smoking status				
Never	61(72.6)	48(73.8)	73(88.0)	0.031
Former/current	23(27.4)	17(26.2)	10(12.0)	
EGFR mutation				
Exon 18	1(1.2)	0	2(2.4)	0.462
Exon 19	36(42.9)	24(36.9)	36(43.4)	
Exon 21	41(48.8)	38(58.5)	36(43.4)	
Unclear	6(7.1)	3(4.6)	9(10.8)	
Systemic therapy				
First-line EGFR TKIs	38(45.2)	30(46.2)	40(48.2)	0.927
Second-line EGFR TKIs	46(54.8)	35(53.8)	43(51.8)	
Lung-molGPA				
1–2	56(66.6)	19(29.2)	28(33.7)	< 0.001
2.5–3	24(28.6)	33(50.8)	41(49.4)	
3.5–4	2(2.4)	12(18.5)	13(15.7)	
Unclear	2(2.4)	1(1.5)	1(1.2)	
Brain metastatic time				
Initial treatment	47(56.0)	41(63.1)	56(67.5)	0.303
In the course of treatment	37(44.0)	24(36.9)	27(32.5)	

*EGFR* epidermal growth factor receptor, *TKI* tyrosine kinase inhibitors

### Survival outcomes for the entire cohort in the study

**Fig. 1:**
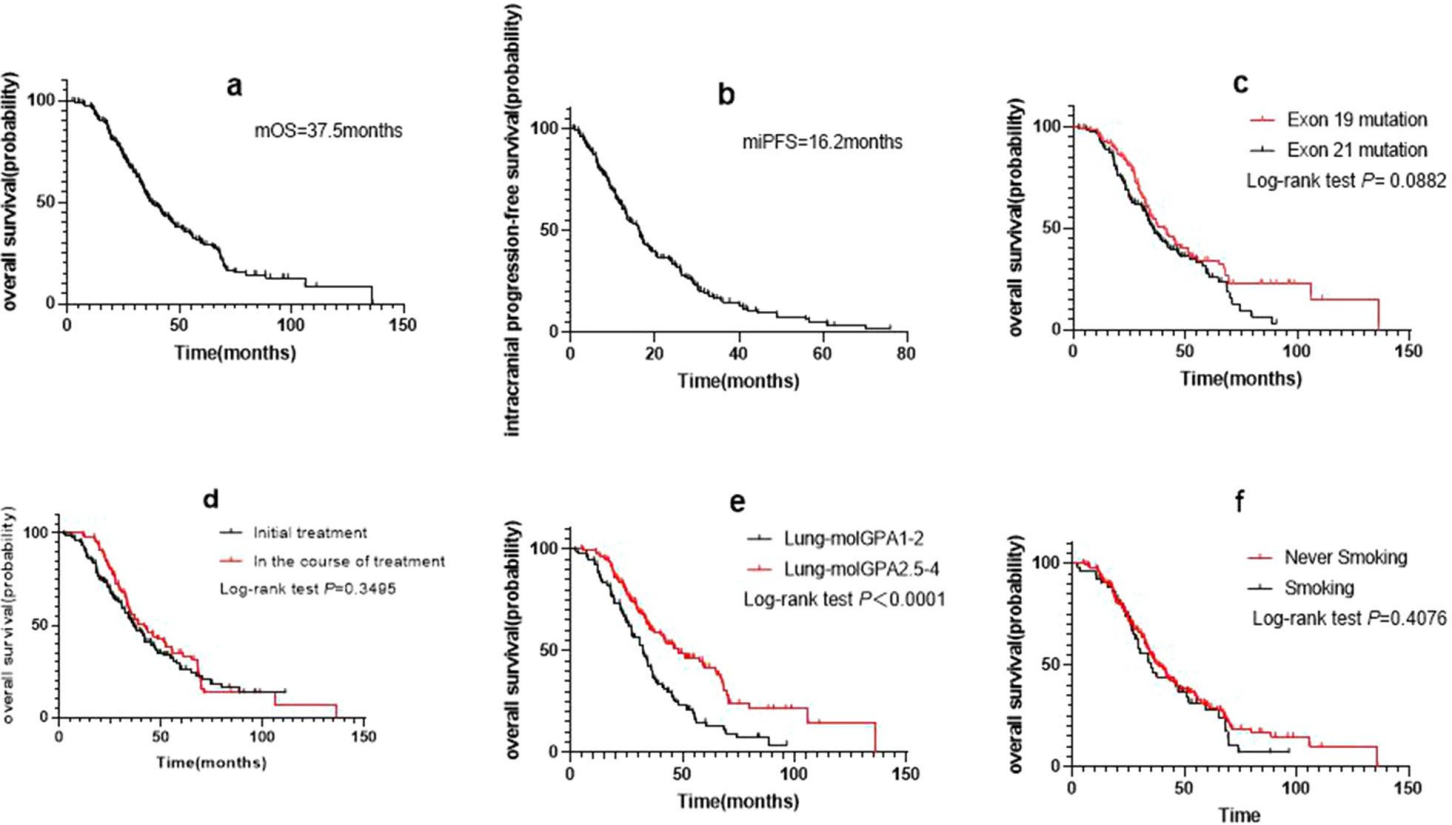
**a** Overall survival (OS) of the entire cohort. **b** Intracranial progression-free survival (iPFS) of the entire cohort. OS of patients stratified according to **c** *EGFR* mutation status, **d** time of occurrence of brain metastasis, **e** Lung-molGPA, and **f** Smoking Status

As of March 2021, 159 deaths (68.5%) were recorded. The median follow-up duration was 60.1 (interquartile range, 48.0 to 90.6) months. Median OS was 37.5 months, while median iPFS was 16.2 months (Fig. 1a, b). There was a trend for difference in OS between groups with mutations in exons 19 and 21 (40.7 months vs. 35.8 months; log-rank *p* = 0.0882; HR: 0.7559; 95% CI 0.5465 to 1.046; Fig. 1c). The median OS in the patients with BM at the initial diagnosis and those with BM during the treatment was 36.1 months and 41.6 months, respectively (log-rank *p* = 0.3495; HR: 1.161; 95% CI 0.8491 to 1.589; Fig. 1d).

The distribution predicted by Lung-molGPA in the three radiotherapy groups was unequal. According to the grading criteria, patients with scores 1–2 were classified into group A, while those with scores 2.5–4 were classified into group B for statistical analysis. The median survival time of patients in groups A and B was 32.3 months and 48 months, respectively (log-rank *p* < 0.0001; HR: 1.925; 95% CI 1.390 to 2.667; Fig. 1e).

### Effects of different radiotherapy modes on iPFS

**Fig. 2:**
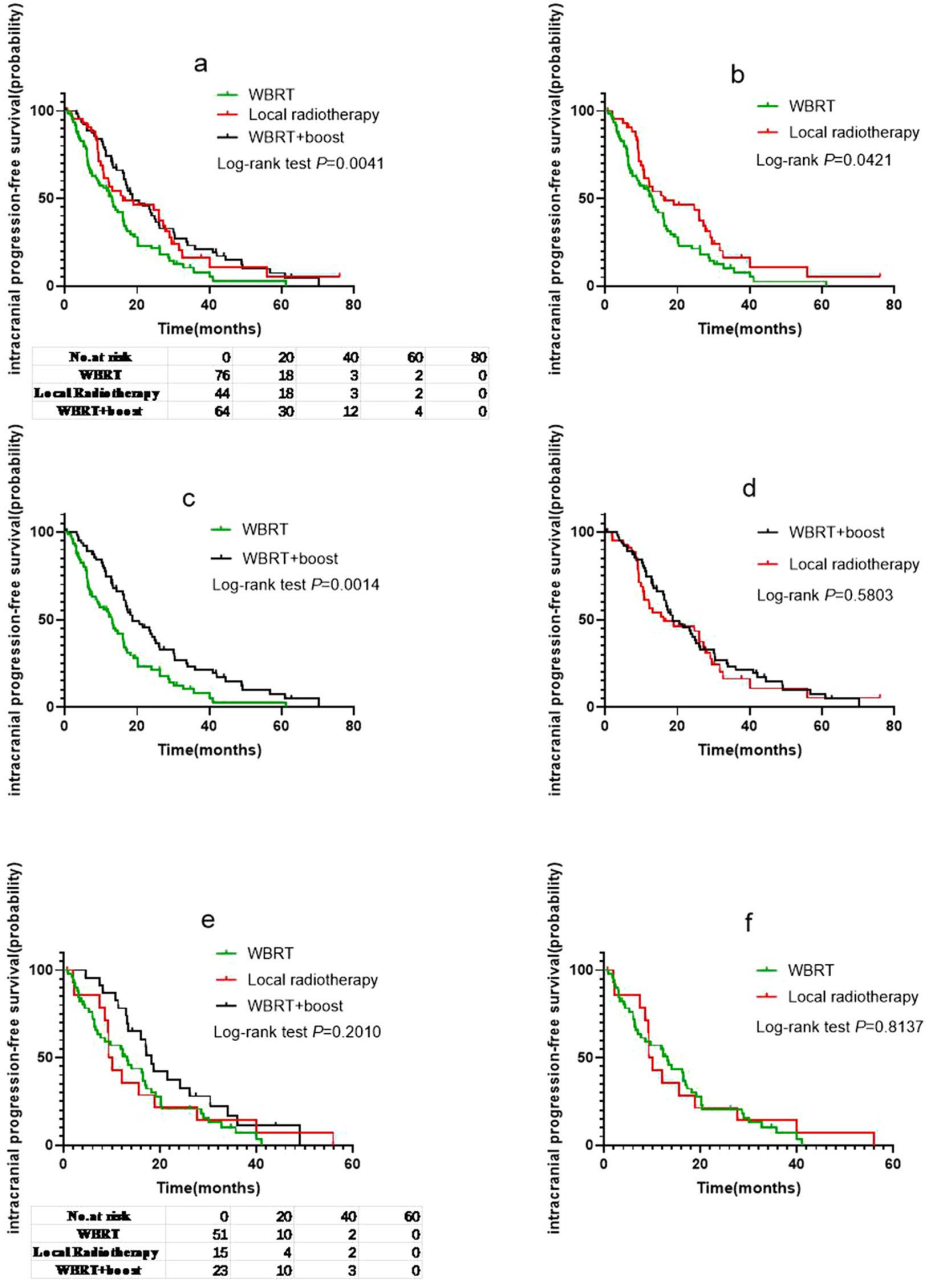

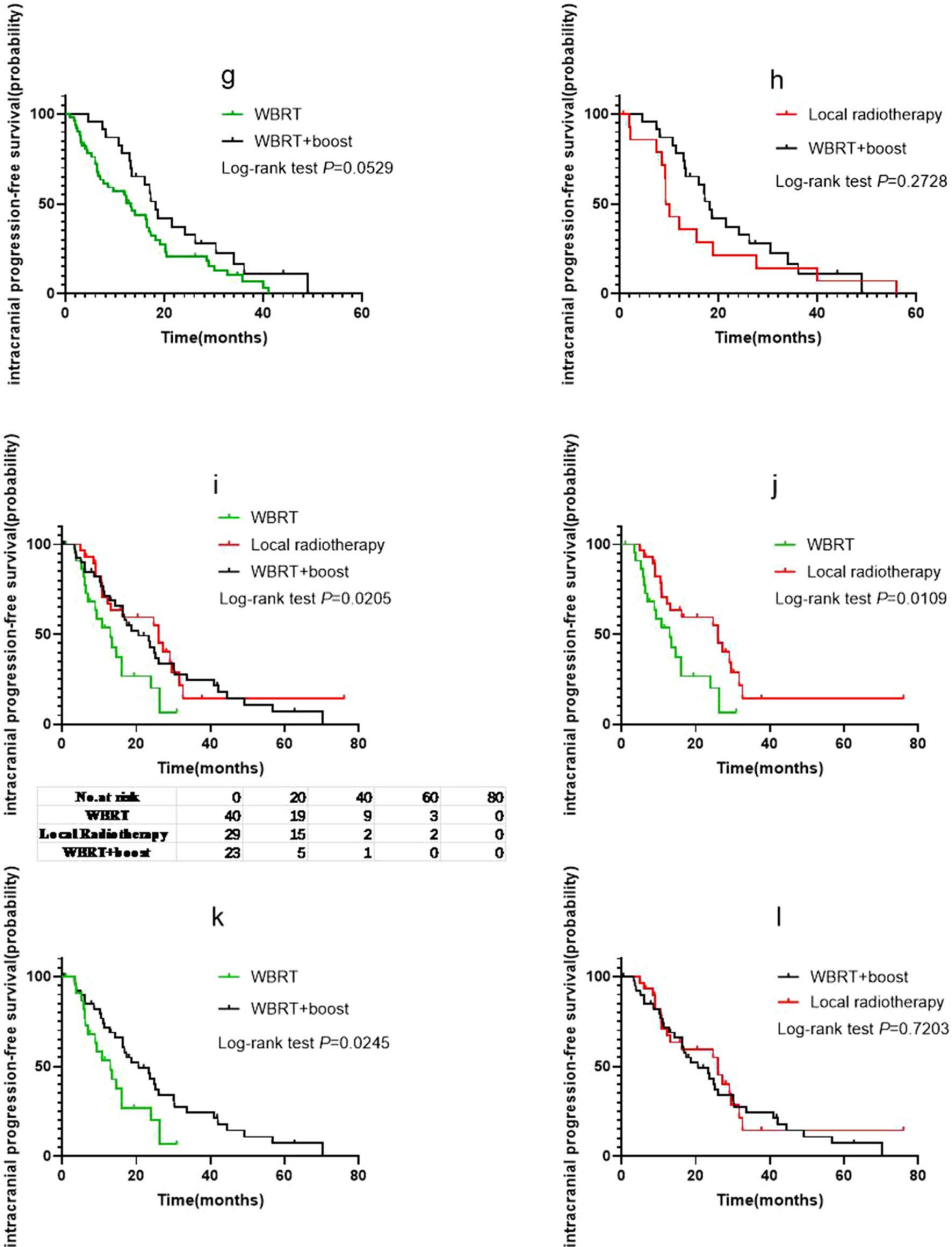
**a** Intracranial progression-free survival (iPFS) of patients according to different the brain radiotherapy strategies. **b** Comparison of iPFS between the whole brain radiotherapy (WBRT) and local radiotherapy groups. **c** Comparison of iPFS between the WBRT and WBRT + Boost groups. **d** Comparison of iPFS between the local radiotherapy and WBRT + Boost groups. **e** iPFS of patients in group A stratified according to different brain radiotherapy strategies and **f**–**h** the differences between them. **i** iPFS of patients in group B stratified according to different brain radiotherapy strategies and **j**–**l** the differences between them. The results showed that in the low Lung-molGPA groups, only WBRT group and WBRT + Boost group trended towards statistically significant, while in the high Lung-molGPA groups, the difference between WBRT group and the other two groups was statistically significant

Of the 232 patients who received radiotherapy, 184 had intracranial progression after radiotherapy (WBRT group, 76; local radiotherapy group, 44; and WBRT + Boost group, 64). The iPFS analysis was performed with WBRT, local radiotherapy, and WBRT + Boost groups. The median iPFS in the WBRT, local radiotherapy, and WBRT + Boost groups was 13, 16.2, and 18.7 months, respectively (Fig. 2a); and, there were significant differences in median iPFS between the WBRT and local radiotherapy groups (*p* = 0.0421; HR: 1.534; 95% CI 1.027 to 2.290; Fig. 2b), and WBRT and WBRT + Boost groups (log-rank *p* = 0.0014; HR: 1.765; 95% CI 1.221 to 2.550; Fig. 2c). Therefore, the iPFS in patients receiving local radiotherapy or WBRT + Boost was better than that in patients receiving WBRT. However, there was no significant difference in iPFS between the local radiotherapy and WBRT + boost groups (log-rank *p* = 0.5803; HR: 0.8861; 95% CI 0.5706 to 1.376; Fig. 2d).

Further, as Lung-molGPA had a significant impact on the prognosis of patients, we performed an iPFS analysis between the three groups stratified by Lung-molGPA score. For patients in the group A, the median iPFS was 13.2, 9.65, and 18.2 months (Fig. 2e). The difference in iPFS between the WBRT and local radiotherapy groups was not statistically significant (log-rank *p* = 0.8137; HR: 1.071; 95% CI 0.5930 to 1.934; Fig. 2f), but that between WBRT and WBRT + Boost groups trended towards statistically significant (log-rank *p* = 0.0529; HR: 1.657; 95% CI 1.010 to 2.718; Fig. 2g). Moreover, there was no significant difference between the WBRT + Boost and local radiotherapy groups (log-rank *p* = 0.2728; HR: 1.438; 95% CI 0.7026 to 2.944; Fig. 2h).

For patients in the group B, the median iPFS of the three groups was 13, 26.1, and 20.6 months (Fig. 2i). The difference between the WBRT and local radiotherapy groups was statistically significant (log-rank *p* = 0.0109; HR: 2.200; 95% CI 1.090 to 4.442; Fig. 2j), and that between the WBRT and WBRT + Boost groups was also statistically significant (log-rank *p* = 0.0245; HR: 1.863; 95% CI 0.9672 to 3.590; Fig. 2k). However, the difference between WBRT + Boost and local radiotherapy groups was not statistically significant (log-rank *p* = 0.7203; HR: 1.106; 95% CI 0.6335 to 1.932; Fig. 2l).

### Effects of different radiotherapy modes on OS

**Fig. 3:**
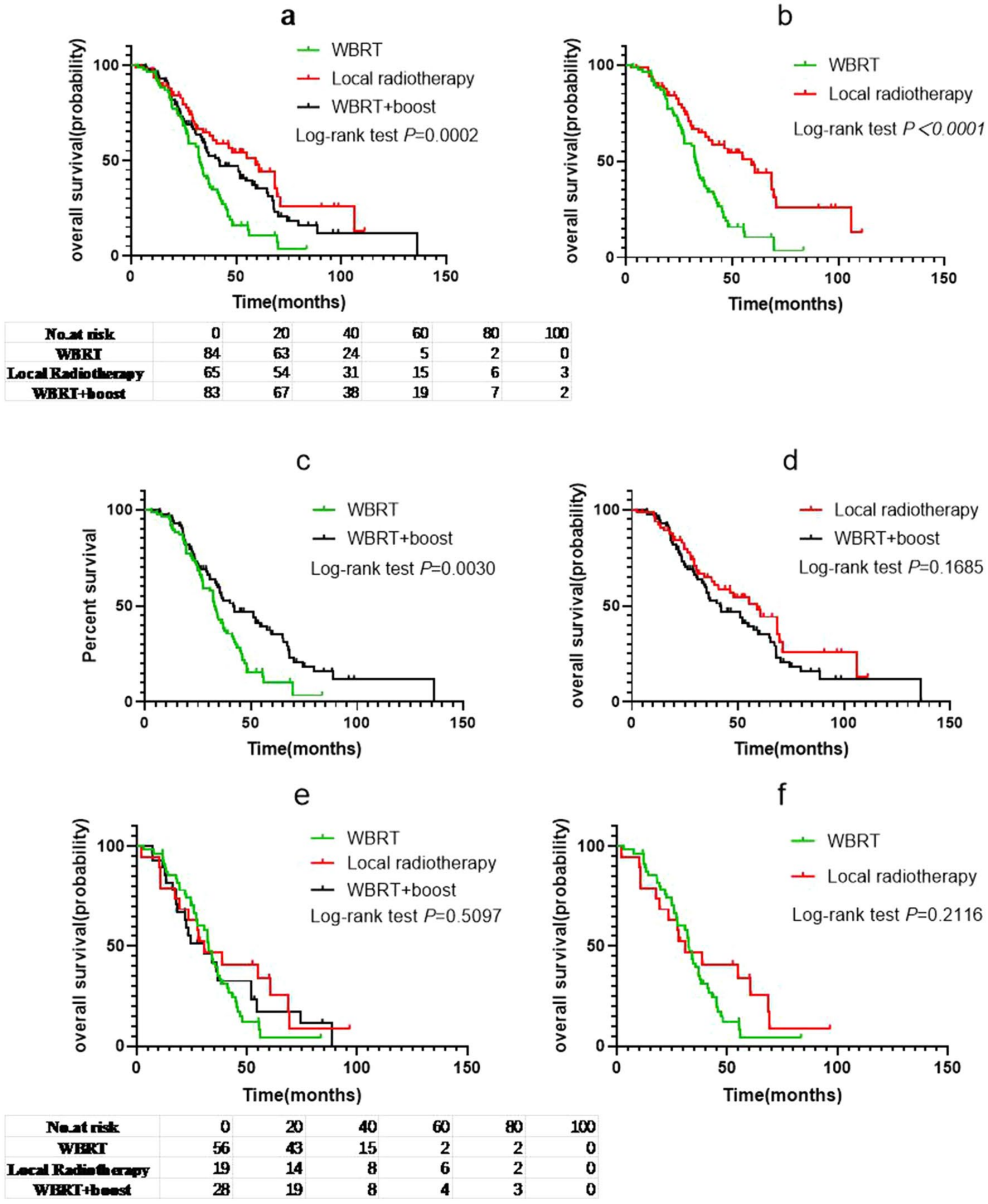

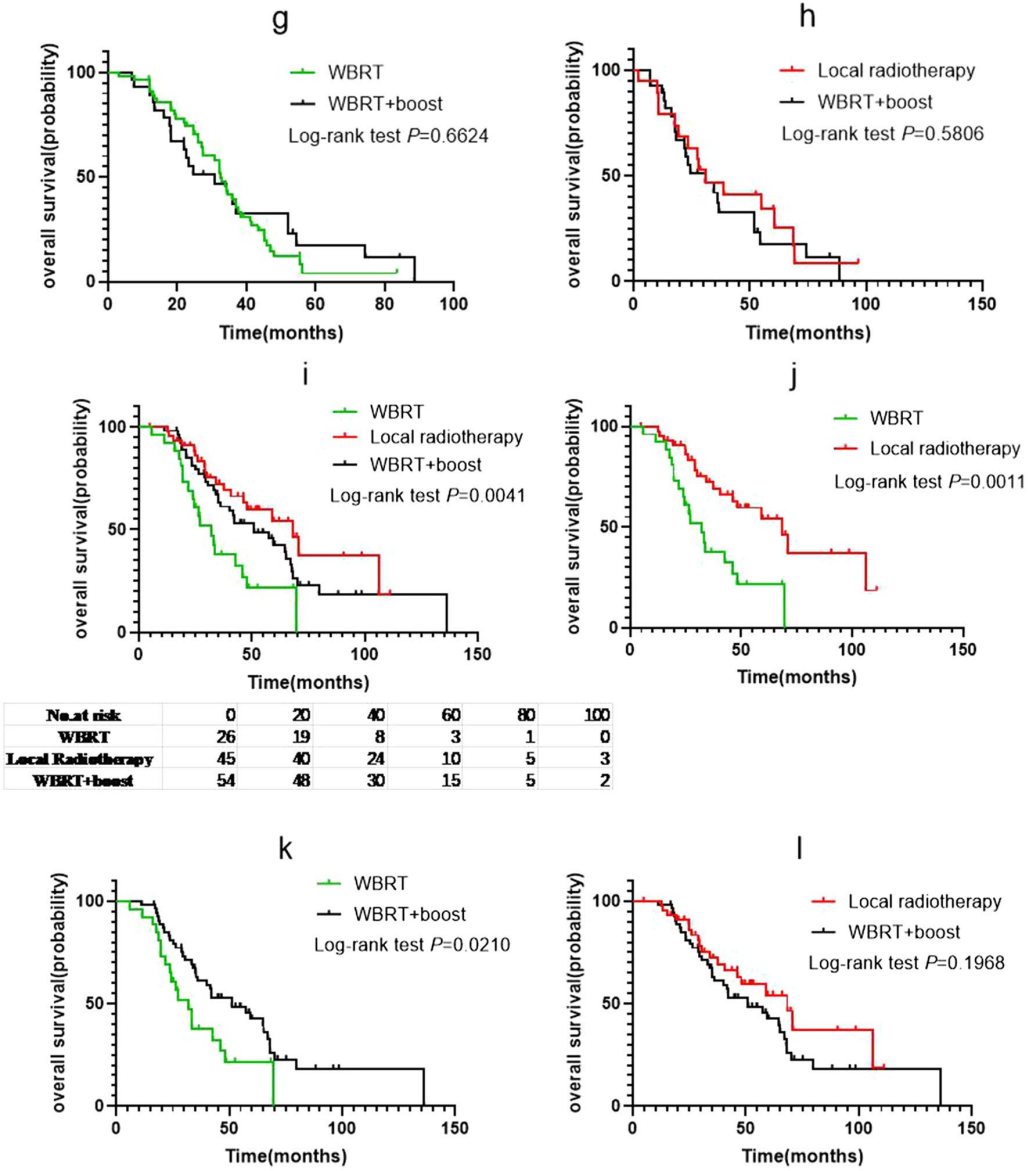
**a** Overall survival (OS) of patients according to different brain radiotherapy strategies. **b** Comparison of OS between the whole brain radiotherapy (WBRT) and local radiotherapy groups. **c** Comparison of OS between the WBRT and WBRT + Boost groups. **d** Comparison of OS between the local radiotherapy and WBRT + Boost groups. **e** OS of patients in group A stratified according to different brain radiotherapy strategies and **f**–**h** the differences between them. **i** OS of patients in group B stratified according to different brain radiotherapy strategies and **j**–**l** the differences between them. The results showed that in the low Lung-molGPA groups, there was no significant difference among the three radiotherapy methods, while in the high Lung-molGPA groups, the difference between WBRT group and the other two groups was also statistically significant

The analysis of OS in patients in the WBRT, local radiotherapy, and WBRT + Boost groups indicated median OS to be 32.8, 59.1, and 41.7 months, respectively (Fig. 3a). There was a significant difference in the median OS between the WBRT and local radiotherapy groups (log-rank *p* < 0.0001; HR: 2.209; 95% CI 1.495 to 3.265; Fig. 3b), and WBRT and WBRT + Boost groups (log-rank *p* = 0.0030; HR: 1.660; 95% CI 1.163 to 2.369; Fig. 3c). However, there was no significant difference in OS between the local radiotherapy and WBRT + Boost groups (log-rank *p* = 0.1685; HR: 0.7458; 95% CI 0.4950 to 1.124; Fig. 3d).

Furthermore, patients were divided into group A and group B according to the Lung-molGPA score. The median OS of the WBRT, local radiotherapy, and WBRT + Boost groups was 32.5, 30.9, and 30.8 months, respectively, for patients in the group A. Moreover, there was no significant difference in median OS between the three brain radiotherapy regimens (log-rank *p* = 0.5097, Fig. 3e). There was also no significant difference between the WBRT and the other two groups (log-rank *p* = 0.2116, HR: 1.422, 95% CI 0.8273 to 2.445, Fig. 3f; and log-rank *p* = 0.6624, HR: 1.115, 95% CI 0.6755 to 1.840, Fig. 3g), and the local radiotherapy and WBRT + Boost groups (log-rank *p* = 0.5806, HR: 0.8333, 95% CI 0.4360 to 1.592, Fig. 3h).

For patients in the group B, there was significant difference in OS between patients receiving different radiotherapy modes (log-rank *p* = 0.0041, Fig. 3i). The median OS of the patients was 32, 68.4, and 51 months in the different groups. The OS of patients receiving local radiotherapy or WBRT + Boost was better than that of patients receiving WBRT-alone. The difference was statistically significant between the WBRT and local radiotherapy groups (log-rank *p* = 0.0011, HR: 2.698, 95% CI 1.319 to 5.520, Fig. 3j), and the WBRT and WBRT + Boost groups (log-rank *p* = 0.0210, HR: 1.878, 95% CI: 0.9917 to 3.557, Fig. 3k). However, there was no significant difference between the WBRT + Boost and local radiotherapy groups (log-rank *p* = 0.1968, HR: 0.6976, 95% CI 0.4091 to 1.190, Fig. 3l).

We further performed univariate and multivariate analysis as shown in Table 3. The univariate analysis results show that the effect of gender, age, smoking status, brain metastatic time on prognosis was not statistically significant. Multivariate analysis showed that only brain radiotherapy mode and Lung-molGPA were independent predictive factors (*p* = 0.004).

**Table 3:** Univariable and Multivariable analyses of covariable associated with OS

	Univariable analysis			Multivariable analysis		
Variable	HR	95% CI	P	HR	95% CI	P
Age (y)						
< 60 vs ≤ 60	0.959	0.684 to 1.344	0.807			
Sex						
Female vs male	1.014	0.733to 1.402	0.935			
Smoking status						
Never vs current/former	1.136	0.786 to 1.643	0.497			
EGFR mutation						
Exon 19	0.721	0.520 to 1.000	0.050	0.800	0.495 to 1.292	0.362
Exon 21	1.252	0.914 to 1.715	0.161	1.104	0.693 to 1.104	0.678
First-line TKI therapy						
Yes v no	1.480	1.077 to 2.032	0.016	1.358	0.985 to 1.872	0.061
Lung-molGPA						
1–2 v 2.5–4	0.513	0.374 to 0.703	< 0.001	0.606	0.433 to 0.849	0.004
Radiotherapy strategies						
WBRT	1.835	1.326 to 2.539	< 0.001	1.529	1.084 to 2.157	0.016
Local radiotherapy	0.456	0.299 to 0.694	< 0.001	0.530	0.340 to 0.827	0.005
WBRT + Boost	0.623	0.434 to 0.894	0.010	0.744	0.509 to 1.087	0.126
Brain metastatic time						
Initial treatment vs In the course of treatment	1.083	0.923 to 1.271	0.329			

## Discussion

The recent evolution of targeted therapies has helped prolong the OS of patients with *EGFR*-mutant advanced lung adenocarcinoma [26–28]. As per a previous study, the median OS was extended to 31.8 months after administration of the first generation EGFR TKIs, while the advent of the third generation EGFR TKIs extended the survival to 38.6 months. [29] However, this enhanced OS increased the probability of developing BM. Moreover, BM frequently occurred in *EGFR*-mutant NSCLC, with approximately 8–49% that occurred at the initial diagnosis and approximately 24% during the treatment course [7, 30]. In the present study, 38.2% of the patients developed BM during the treatment, emphasizing the importance of local treatment, such as brain radiotherapy, in patients who develop BM after systemic treatment [17]. To the best of our knowledge, the present study is the first to compare the effects of three radiotherapy modes for BM on the prognosis of patients with *EGFR*-mutant NSCLC.

The number of craniocerebral metastases is closely related to the prognosis of patients with NSCLC, which plays an important role in the formulation of brain radiotherapy strategy. In clinical practice, the formulation of radiotherapy regimens is primarily based on the number of BM. For example, in patients with > 3 metastatic foci, WBRT was the main treatment; but, in patients with ≤ 3 metastatic foci, local radiotherapy was selected [14]. In addition to the number of BM, recent studies have found that Lung-molGPA offers a better predictive ability and may affect decisions on the brain radiotherapy strategy [27]. This suggests that Lung-molGPA can comprehensively and accurately reflect the prognosis in patients with NSCLC with BM. In the present study, we divided the patients into two groups according to the Lung-molGPA score. Survival analysis showed that the median OS in patients in the high score group was significantly longer than that in patients in low score group (log-rank *p* < 0.0001), which was consistent with the results reported previously [27].

The proportion of patients with low scores in the WBRT group was higher (66.6%), while that of patients with high scores in the local radiotherapy and WBRT + boost groups was 69.3% and 65.5%, respectively, indicating that the difference in the Lung-molGPA constitution ratio existed in different radiotherapy groups. To better clarify the effects of different modes of radiotherapy on the prognosis of patients, we divided the patients into two groups according to the Lung-molGPA score as the high score and low score groups. For patients with low score, there is no statistically difference of iPFS among WBRT + boost group, local radiotherapy group and WBRT group. However, the enhanced iPFS failed to translate to improved OS. Therefore, in patients with worse clinical conditions, the synchronous or sequential dose addition based on WBRT has little effect on the increase in OS. In contrast, it may indicate greater side effects that may have an adverse impact on the quality of life [31]. Therefore, it may be more reasonable for such patients to receive radiotherapy with less side effects only for treatment of metastatic lesions. A recent study suggested that the benefit of treatment with local radiotherapy is independent of the number of BM [32] Another study has shown that local radiotherapy can also achieve good local control and prognosis in patients with 5–10 BM [33]. Additionally, we emphasize the importance of systemic treatment in patients in whom the third generation of EGFR TKIs, such as osimertinib, have been widely used with a good effect on both intracranial and extracranial lesions [34, 35].

In patients with high Lung-molGPA score and better clinical condition, survival analysis showed that median iPFS in the local radiotherapy and WBRT + boost groups was longer than that in WBRT group. Moreover, there was no significant difference in iPFS between the local radiotherapy and WBRT + boost groups. The results of the OS were similar to that of iPFS. The OS in the WBRT + boost and local radiotherapy groups was better than that in WBRT group, but there were no significant differences between the WBRT + boost and local radiotherapy groups. These results suggest that the OS of patients with good prognosis is longer, and increasing the local control can further prolong the OS in patients. However, OS in the WBRT + boost high dose group with craniocerebral metastasis was not superior to that in local radiotherapy group, indicating that in patients with good prognosis, the formulation of radiotherapy strategy should consider both local control and side effects of brain radiotherapy. The WBRT + boost intervention may increase toxicity and side effects after brain radiotherapy that may adversely affect the prognosis in patients [31]. Patients with higher scores have a good overall prognosis, and EGFR TKIs are effective against BM [36]. Therefore, results of the present study suggest that the range of radiation target should be reduced in patients with higher Lung-molGPA scores and local radiotherapy should be administered on priority to avoid neurotoxicity of WBRT [18, 19].

The study has some limitations. First, it was a single-center, retrospective study with limited sample size, and thus, had selection bias. Second, there were differences in the type, timing, combination, and sequence of chemotherapy and targeted therapy administered in patients in the study; therefore, it was impossible to evaluate the effects of the drugs on patients. Third, the uneven dose of radiotherapy in the same group may have an impact on the prognosis of patients to some extent. Therefore, considering these limitations, we warrant a large sample size, multi-institutional, prospective study to confirm the findings of the present study.

## Conclusion

The present study showed that in patients with *EGFR*-mutant lung adenocarcinoma with BM, local radiotherapy and WBRT + Boost perform similarly well both in the subgroups with low and high scores of Lung-molGPA. Considering the side effect caused by whole brain radiotherapy, we recommended local radiotherapy as optimal brain radiation mode for those subtype lung cancer patients. Prospective study will be performed to verify the findings in the study.

## Data Availability

Data are available on request to the authors.
