# Correction to: Effect of brain radiotherapy strategies on prognosis of patients with *EGFR*-mutant lung adenocarcinoma with brain metastasis

**DOI:** 10.1186/s12967-021-03175-9

**Published:** 2021-12-07

**Authors:** Guangchuan Deng, Yingyun Zhang, Jiaojiao Ke, Qi Wang, Hongyue Qin, Jianbin Li, Zhenxiang Li

**Affiliations:** 1 Department of Radiation Oncology, Shandong Cancer Hospital and Institute, Shandong First Medical University and Shandong Academy of Medical Sciences, Jinan, 250117 China; 2 Shandong First Medical University and Shandong Academy of Medical Sciences, Jinan, People’s Republic of China; 3 Weihai Central Hospital, Weihai, People’s Republic of China

## Correction to: J Transl Med (2021) 19: 486https://doi.org/10.1186/s12967-021-03161-1

In the original publication [1] there was an incorrect funding section. The incorrect and correct funding information is published in this correction article. The original article has been updated.

### Incorrect funding

None.

### Correct funding

Taishan Scholars Program of Shandong Province (NO.ts 20190982).
